# Efficient exciton generation in atomic passivated CdSe/ZnS quantum dots light-emitting devices

**DOI:** 10.1038/srep34659

**Published:** 2016-09-30

**Authors:** Byoung-Ho Kang, Jae-Sung Lee, Sang-Won Lee, Sae-Wan Kim, Jun-Woo Lee, Sai-Anand Gopalan, Ji-Sub Park, Dae-Hyuk Kwon, Jin-Hyuk Bae, Hak-Rin Kim, Shin-Won Kang

**Affiliations:** 1 Center for Functional Devices Fusion Platform, Kyungpook National University, Sankyuk-dong, Bukgu, 702-701 Daegu, Republic of Korea; 2 School of Electronics Engineering, College of IT Engineering, Kyungpook National University, Sankyuk-dong, Bukgu, 702-701 Daegu, Republic of Korea; 3 Department of Electronic Engineering, Kyungil University, Buho-ri, Hayang-eup, 712-701 Gyeongsan-si, Republic of Korea

## Abstract

We demonstrate the first-ever surface modification of green CdSe/ZnS quantum dots (QDs) using bromide anions (Br^-^) in cetyl trimethylammonium bromide (CTAB). The Br^-^ ions reduced the interparticle spacing between the QDs and induced an effective charge balance in QD light-emitting devices (QLEDs). The fabricated QLEDs exhibited efficient charge injection because of the reduced emission quenching effect and their enhanced thin film morphology. As a result, they exhibited a maximum luminance of 71,000 cd/m^2^ and an external current efficiency of 6.4 cd/A, both significantly better than those of their counterparts with oleic acid surface ligands. In addition, the lifetime of the Br- treated QD based QLEDs is significantly improved due to ionic passivation at the QDs surface.

Quantum dot light-emitting device (QLED) has received significant attention as a potential component of next-generation displays that may replace liquid crystal display (LCD) and organic light-emitting diode (OLED) systems. The advantages of quantum dots (QDs) in electroluminescence (EL) devices include the possibility of tuning the emission color by changing the QD size and enhanced color purity with a full-width at half-maximum (FWHM) peak, as narrow as 18 to 30 nm in the visible range^1^. In addition, solvent processing can be simplified via spin-casting or contact printing, either of which allow the QLED to meet a broad range of standards set by the National Television System Committee (NTSC)^2^,^3^.

Existing QLEDs are built using the traditional architecture of an OLED, where a solvent process is combined with a vacuum process, and are often comprised of materials such as organic monomers or metal oxides. Sun *et al*. reported on a QLED with a hybrid structure that incorporates Alq_3_ as its electron transport layer (ETL) and showed that the optimized ETL, formed via thermal evaporation, resulted in high QLED performance^4^. There have been several proposals regarding the composition of QLEDs, such as the use of nickel oxide (NiO) and zinc oxide (ZnO):tin oxide (SnO_2_) thin films for the metal-oxide charge transport layer and the use of molybdenum oxide (MoO) in an inverted structure^5^,^6^. These studies investigated the energy transfer phenomenon in the EL recombination zone within the QDs and resulted in QLEDs with good performance. However, they employed vacuum evaporation for deposition of the ETL and the hole transport layer (HTL). Recently, metal-oxide nanoparticles (NPs) with high electron mobilities have been used in ETLs in order to enhance device performance without using a vacuum process. Kim *et al*., Qasim *et al*., Qian *et al*., and Madners *et al*. reported on QLEDs manufactured using alcohol dispersed metal-oxide NPs, such as titanium oxide (TiO_2_) and ZnO NPs, as the ETL, and confirmed the possibility of using solution processing to fabricate the ETL^7^,^8^,^9^,^10^. These studies indicated high-efficient device performance and showed well defined energy transfer between the metal-oxide NPs and the cathode. Various investigations of the QLED structure and charge transport materials have been reported. As mentioned, QDs may be used in the emissive layer (EML). In this context, it is important to use surface interfacial control to produce QLEDs with very high performance. In an effort to improve exciton-involved radiative recombination in QLEDs, Shen *et al*. and Lim *et al*. reported QLEDs prepared by controlling the shell thickness of core/shell heterostructured QDs. The luminance and luminescence efficiency of the optimized QLEDs improved significantly^11^,^12^. In addition, in order to enhance the efficiency of QDs, several researchers have focused on surface modulation using various organic ligands^13^,^14^,^15^. For instance, Shen *et al*. reported synthesizing high-efficiency blue-violet QLEDs by using shorter 1-octanethiol ligands on QDs. This report shows improvements in the overall charge balance of the QLEDs and increased luminance efficiency^16^. Nevertheless, these studies implemented long-chain modifications, which can reduce the efficiency of charge transport from neighboring layers. In order to improve the charge transport, some reports suggested a new strategy that inorganic halogen ions are role of short-chain ligand on QDs^17^,^18^,^19^. Therefore, inspection of QLEDs, reducing the interparticle spacing (IPS) of CdSe/ZnS QDs with short-chain ligands is necessary to enhance the exciton generation rate in solid-state CdSe/ZnS QDs emissive layer.

In the present study, we demonstrate the effects of using bromide anions (Br^-^) as short inorganic halogen ligands on green CdSe/ZnS QDs via cetyl trimethylammonium bromide (CTAB) treatment, and coordination of the Br^-^ on the CdSe/ZnS QDs film via nucleophilic substitution^20^. This study constitutes the first-ever report on the effects of CTAB treatment on the interface between QDs and a neighboring layer. In this study, the Br^-^ in CTAB led to reduction of the QD IPS and an increase in charge transport from neighboring layers. To evaluate the performance of QLEDs with Br^-^, we fabricated solution-processible QLEDs using modified versions of existing fabrication methods^21^,^22^,^23^. The resulting Br^-^-capped QLED exhibited significantly higher performance than its counterpart consisting of OA organic long-chain ligands. To the best of our knowledge, our modification of the QD surface ligand results in the largest reported improvement in QLED performance of surface modified QDs over that of organic ligand capped QDs.

## Results

### QD surface modulation via CTAB treatment

**Figure 1:**
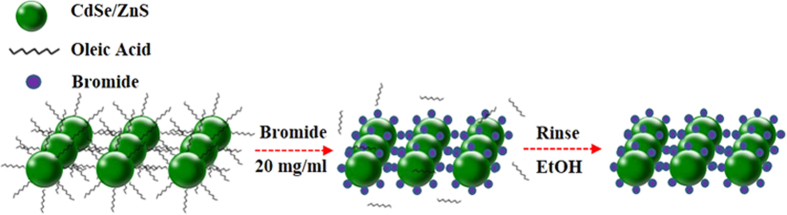
Schematic diagram of the surface modulation from OA to Br^-^ by CTAB treatment.

**Figure 2:**
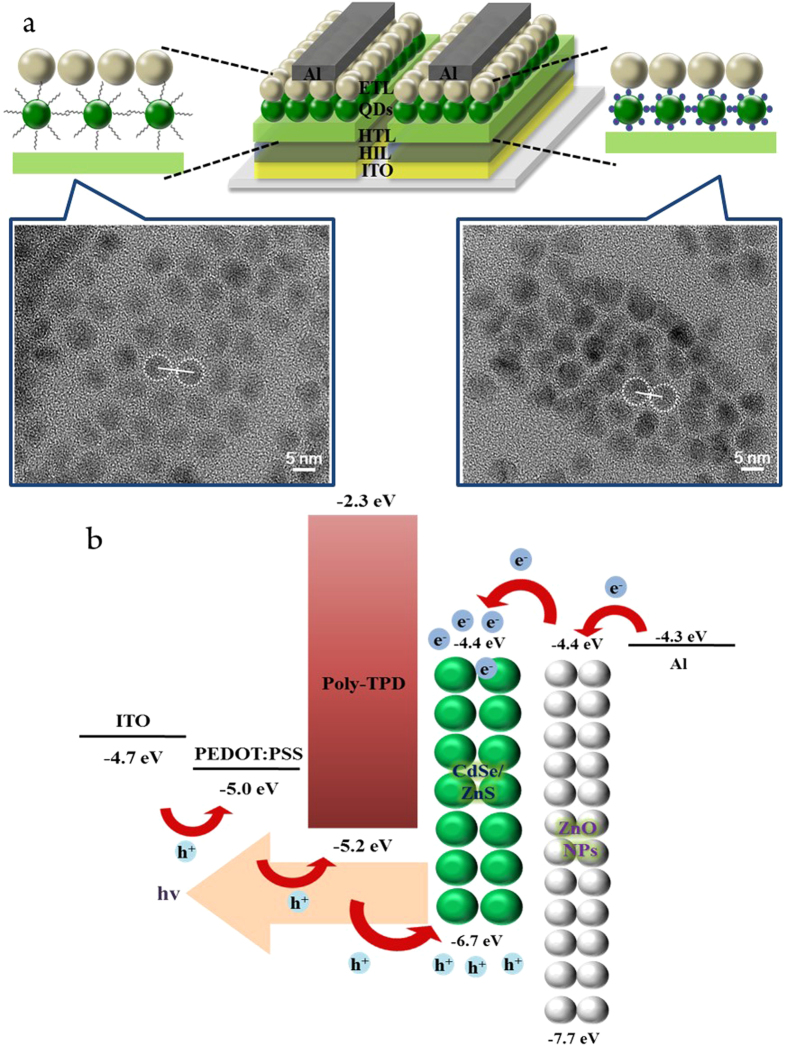
Schematic diagram of QLEDs structure. (**a**) Detailed QLED structure and QDs interparticle spacing in EML (scale bar: 5 nm). (**b**) Energy band diagram.

The exchange of ligands from OA to Br^-^ was performed by treating the CdSe/ZnS QDs solid-state film with a 20 mg/ml CTAB solution after vacuum thermal annealing. In this procedure, the ammonium cation was combined with the deprotonated OA via nucleophilic substitution, and was subsequently removed by rinsing with an alcohol solution, as shown Fig. 1. The schematic and band diagram of the QLEDs and the Br^-^ containing EML are shown in Fig. 2.

Generally, OA-capped CdSe/ZnS QDs have chains of approximately 1.7 nm in length, with 18 carbon bonding. This long-chain ligand increases the number of charge-trapping sites and reduces the EML film packing density because of the large QD IPS. However, in the proposed surface modification, the inorganic Br^-^ anions were coordinated to the Zn^2+^-rich QD surface and the ammonium-OA complex was rinsed with ethanol. Consequently, the QD surface has only Br^-^ anions bound to it, and the organic ligands have been removed. As mentioned before, reducing the IPS is essential to formation of a close-packed EML and to effective confinement of charges from adjacent layer in the QD EML. If the IPS is reduced via surface ligand exchange, the packing density of the QD EML can increase. This correlation between IPS and packing density can be derived from Kuwabara’s cell model, which is used to calculate the IPS in a particulate system^24^. The IPS of a particulate system is the distribution of distances between QDs contained within a given volume and is expressed by the following equation (1).


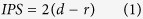


where *r* is the radius of the CdSe/ZnS QDs and *d* is the radius of the CdSe/ZnS QDs with surface ligands, as shown in Fig. 2a. In this case, we confirmed that the Br-capped QDs exhibited close center-to-center pitch than the OA-capped QDs because of the reduced IPS. Thus, Br^-^ ligand exchange can increase the close-packed film state. In addition, as shown in Fig. 2b, short chain QD ligands can also enhance the exciton energy-transfer rate from an adjacent charge transport layer to QD layer^25^. This exciton energy-transfer rate (EETR) can be expressed using the following equation (2).


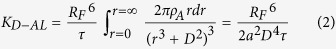


where *a* is the QD radius, *R*_*F*_ is the Föster radius, *D* is the distance from a donor to a plane of QD acceptors, and *τ* is the donor PL relaxation time. In equation (2), the EETR is dependent on the QD radius and distance between the adjacent layer and the QD EML. Thus, the EML film’s packing density and efficiency of charge confinement increase because Br^-^ is smaller than the OA, as summarized in Table 1.

### Optical properties of QDs

**Figure 3:**
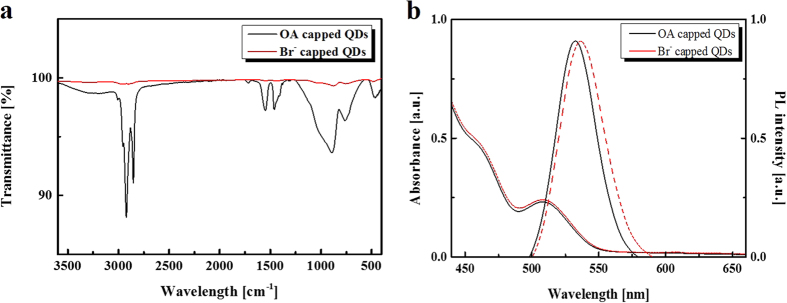
The materials characterization of bromide anion modulation on CdSe/ZnS quantum dots. (**a**) FT-IR spectra of OA-capped CdSe/ZnS QDs and Br^-^-capped CdSe/ZnS QDs. (**b**) The optical properties of CdSe/ZnS QDs by CTAB treatment.

In order to verify coordination of the Br^-^ anions to the CdSe/ZnS QD surfaces, we analyzed the materials’ Fourier transform infrared (FT-IR) spectra. As shown in Fig. 3a, the OA-capped CdSe/ZnS QDs have peaks at wavenumbers (1/λ) of approximately 2,921 cm^−1^ and 2,852 cm^−1^ from of C-H bonding, 1,548 cm^−1^, and 1,458 cm^−1^ from COO^-^ bonding, and 890 cm^−1^ from C=C bonding. However, no organic peaks are observed for the Br-capped CdSe/ZnS QDs. The proportion of Br^-^ in the Br-capped CdSe/ZnS QDs is calculated using energy dispersive spectroscopy (Supplementary information Fig. S1). These results confirm that the Br^-^ is well modulated on the QD surface via electrostatic interaction. Subsequently, the QD packing density was determined; this measurement is essential because non-uniformity in a solid state film can induce device performance degradation. To verify its packing density, atomic force microscopy (AFM) was used to measure the film’s surface roughness. The Br-capped QD film had a more homogeneous morphology than the OA-capped QD film because of its smaller IPS (Supplementary information Fig. S2). Figure 3b shows the absorption and photoluminescence (PL) spectra of the OA and Br-capped CdSe/ZnS QD films. Both absorption spectra are similar, but the PL spectrum of the Br-capped CdSe/ZnS QDs is slightly red shifted by approximately 5 nm, and has a higher FWHM, than the OA-capped CdSe/ZnS QDs. This effect may stem from three possible causes^26^,^27^,^28^: (i) stronger electronic coupling among the neighboring QDs via extension of the electron wave function outside the QDs; (ii) charge transfer between the QDs and the surface ligands which delocalizes the electron wave function within the QDs and relaxes the quantum confinement effect; and (iii) increase in the red shift as the IPS decreases, which indicates delocalization of the excitonic wave function and the formation of extended states. Thus, we think that stronger electron coupling, effective charge transfer, and decreasing IPS are the main factors contributing to the red shift observed in the emission from the CTAB treated QD film.

The relative photoluminescence quantum yield (PL QY) of the QDs was measured by comparing their PL intensities with those of a primary standard dye solution (Rhodamine 6G) at the same optical density (0.05), at the 450 nm excitation wavelength^29^. The Br-capped QDs exhibit a higher PL QY than the OA-capped QDs, as shown in Table 1 and Supplementary information Fig. S3. The high PL QY indicated the reduction of nonradiative decay in high-quality Br-capped QDs. These results show that Br^-^ anion treatment results in a more efficient thin film emissive layer than is achieved with OA-capped QDs.

### Performance of QLEDs

**Figure 4:**
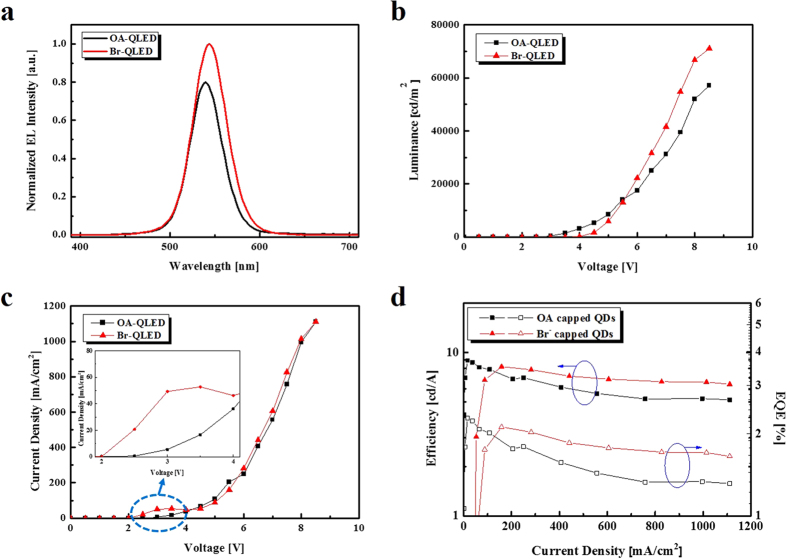
Performance evaluation results for the fabricated QLEDs. (**a**) Normalized EL spectra. (**b**) Voltage-luminance. (**c**) Voltage-current density (inset: enlarge scale of the 2.0–4.0 V). (**d**) Current efficiency-EQE alongside the current density.

The effectiveness of the Br^-^ modified QD film was confirmed by measuring the luminance, current density and external current efficiency-external quantum efficiency (EQE) alongside the current density in the QLEDs. In order to evaluate the efficiency of charge confinement of the Br^-^ surface modification of the QDs, we fabricated two sets of QLEDs: the OA-capped QD based QLED (OA-QLED) and the Br-capped QDs based QLED (Br-QLED). Figure 4 shows the performance of the fabricated QLEDs with an applied voltage of 7 V. The EL intensities of the OA-QLED and the Br-QLED are approximately 540 nm, and no parasitic emission is observed from the adjacent layers. However, Br-capped CdSe/ZnS QDs show a wavelength shift of 4 nm and broadening FWHM due to decreased IPS, as mentioned earlier. Despite being driven by the same applied voltage, the EL intensity of the Br-QLED is much higher than that of the OA-QLED. This shows that Br-capped CdSe/ZnS QDs can enhance the EL intensity by having fewer trap sites than their organic counterparts. Therefore, Br-capped CdSe/ZnS QDs can induce balanced carrier injection, enhanced recombination, and reduced emission quenching. A maximum luminance of 57,000 cd/m^2^ and current efficiency of 5.1 cd/A at maximum luminance are obtained from the OA-QLED. However, the Br-QLED shows superior performance, with a maximum luminance of 71,000 cd/m^2^ and current efficiency of 6.4 cd/A at maximum luminance. Consequently, the Br-QLED exhibits approximately 25% better luminance and current efficiency than the OA-QLED. The Br-QLED has higher luminance than its counterpart at the same current density, as summarized in Table 1. In addition, the current density-voltage (J-V) characteristics of the OA-QLED and Br-QLED are compared in Fig. 4c. The Br-QLED shows higher a space-charge limited current (SCLC) region at the threshold voltage (V_th_) than the OA-QLED. These results demonstrate that the short-chain Br- ligands on the QD surface produce more efficient energy transfer than the long-chain organic OA ligand, without the presence of charge trapping sites in the EML. Therefore, the luminance conversion characteristics of the EML depend on the IPS and the charge trapping sites between adjacent QDs and neighboring layers.

In contrast, as shown in Fig. 4, in the low current density region, the V_th_ of the Br-QLED is slightly higher and the current efficiency-EQE with current density is lower than with the OA-QLED. Through surface modification, the QD IPSs decrease and their packing density increase, which increases the dielectric constant and capacitance of the QD layer^30^,^31^. At the same applied voltage, the net electric field applied to the QD layer is diminished, which augments the V_th_ of Br-QLED. When the applied voltage is higher than V_th_, the luminance and efficiency are dramatically increased and stable because of the effective charge balance without nonradiative recombination in the EML. In order to investigate the effect of surface modification with specific organic chains, we tested trioctylphosphine (TOP) capped QDs and also used CTAB as a solid-state film. In this case, the luminance and efficiency of the QLED decreased due to low nucleophilic substitution reactivity (Supplementary information Fig. S4).

### Lifetime characteristics of QLEDs

**Figure 5:**
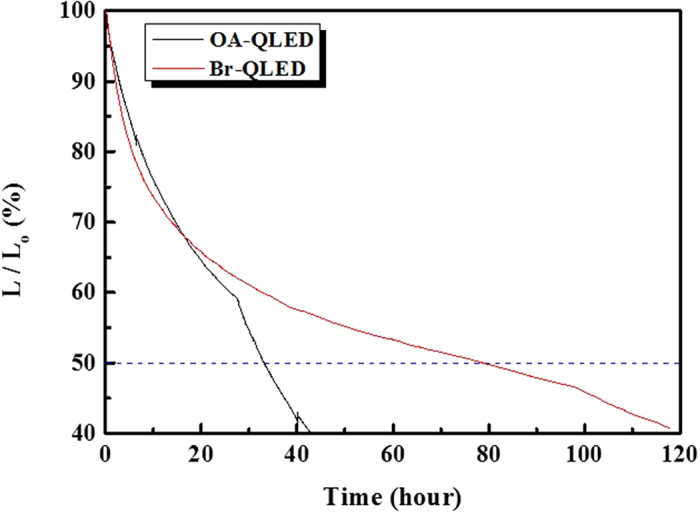
Lifetime characteristics of OA-QLED and Br-QLED without encapsulation. Lifetime characteristics of the OA-QLED (black line) and Br-QLED (red line) at the initial luminance of 900 cd/m^2^, under constant current operation 4 mA at room temperature.

Lifetime tests of the un-encapsulated QLEDs were performed by operating the device at a constant current of 4 mA, and are shown in Fig. 5. All lifetime tests were performed under ambient conditions. The lifetime T50 (measured in h), is the time required for the luminance to decrease to 50% of its initial value. The OA-QLED shows rapid deterioration from an initial luminance of 900 cd/m^2^ within 33 h of continuous operation. In contrast, the brightness of the Br-QLED decays slowly to half of its initial value after 79 h. Its initial luminance is the same as that of the OA-QLED. It is obvious that the device with Br-capped QDs is more stable under continuous operation, and that its lifetime is almost 2.4 times longer than device made with the OA-capped QDs. The improvement in device stability is congruent with the efficient charge confinement in the EML.

### CIE coordinates analysis

**Figure 6:**
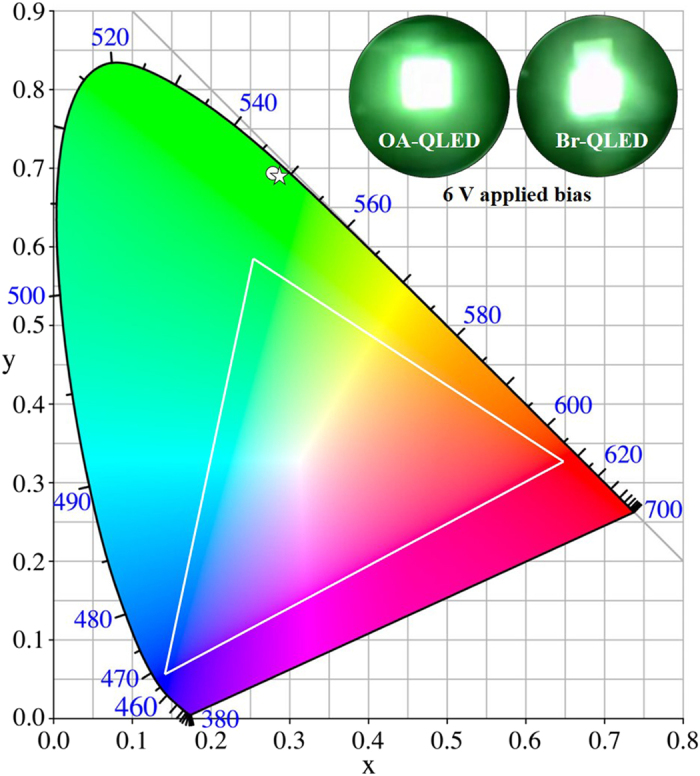
CIE coordinates of the fabricated QLEDs. CIE coordinates of the OA-QLED (circle) and Br-QLED (star) with the NTSC colour triangle. Insets show photographs of EL emission from the QLEDs.

To verify the emission color of the fabricated device, we evaluated the Commission Internationale de Eclairage (CIE) color space for the OA-QLED and the Br-QLED, as shown in Fig. 6. All of the QLEDs display saturated green color coordination, which resides outside the National Television System Committee (NTSC) color triangle. Despite the surface modification, these results show that the color produced is not affected by the difference between Br^-^ anion and OA-capping on the surfaces of the CdSe/ZnS QDs. The inset shows photographs of OA-QLEDs and Br-QLEDs operating with an emission area of 9 mm^2^.

## Discussion

In summary, we demonstrate the use of Br-capped CdSe/ZnS QDs to improve the overall performance of solution-processable QLEDs. Because of their lower charge trapping density, QLEDs with Br-capped QDs not only have enhanced thin film morphology, but also improved luminance and current efficiency compared to QLEDs with OA-capped QDs. Thus, Br-capped QDs provide efficient charge confinement in the EML due to reduced emission quenching, and significantly improved luminance and current efficiency.

## Methods

### Synthesis of gradient CdSe/ZnS quantum dots

Green CdSe/ZnS QDs with chemical-composition gradients were prepared using a method reported in the literature^9^,^32^. For a typical synthesis, 0.1 mmol of cadmium oxide (CdO), 4 mmol of zinc acetate (Zn(CH_3_CO_2_)_2_) and 5 ml of oleic acid (OA) were placed in a 50 ml flask and heated to 150 °C in flowing high-purity Ar gas for 30 min. Then 15 ml of 1-octadecene (ODE) was added to the 3-neck flask and the temperature increased to 300 °C. A stock solution containing 0.2 mmol of selenium (Se) and 3 mmol of sulfur (S) in 2 ml of trioctylphosphine (TOP) was quickly injected into the 3-neck flask. The reaction temperature was maintained for 10 min and then cooled to room temperature. Then, the synthesized QDs were purified by adding toluene, ethanol solution. The mixed solution was centrifuged at 3,000 rpm for 10 min to separate the QDs via precipitation. The supernatant liquid phase was decanted to remove the excess reagent. Subsequently, the QDs were re-dispersed in a non-polar toluene solution (10 mg/ml). The UV-Vis spectrum, PL spectrum and transmittance electron microscopy (TEM) images of the synthesized QDs (Supplementary information Fig. S5).

### Device Fabrication

The QLEDs that were fabricated consisted of an ITO anode (150 nm), a hole injection layer (HIL) (25 nm), a hole transport layer (HTL) (45 nm), a CdSe/ZnS QDs EML (20 nm), an electron transport layer (ETL) (35 nm), and an Al cathode (150 nm). The HIL was formed using poly(ethylenedioxythiophene):polystyrene sulfonate (PEDOT:PSS, Baytron P AI 4083, H. C. Starck) on a patterned ITO substrate via spin coating and drying at 150 °C for 10 min. The HTL was formed using poly(N,N’-bis(4-butylphenyl)-N,N′-bis(phenyl)benzidine (poly-TPD, ADS 254BE, American dye source), is a good resistor for non-polar organic solvents such as toluene, and was dissolved in chlorobenzene to 0.5 wt%. After spin coating, the HTL was dried at 110 °C for 30 min under vacuum conditions. The QDs were dissolved in a toluene solution and spin-coated to form the EML. Annealing was performed for 30 min at 80 °C under vacuum conditions. The ZnO NPs solution was directly coated on the EML at 90 °C for 30 min under vacuum conditions to form the ETL. The ZnO NPs were synthesized via the sol-gel method (Supplementary information Fig. S6). Subsequently, the emissive area of the fabricated device was defined to 9 mm^2^. The characteristics of the fabricated QLEDs were measured using a computer-controlled voltmeter (Keithley 2400s source-meter) and a luminance meter (CS-100A).

## Additional Information

**How to cite this article**: Kang, B.-H. *et al*. Efficient exciton generation in atomic passivated CdSe/ZnS quantum dots light-emitting devices. *Sci. Rep*. **6**, 34659; doi: 10.1038/srep34659 (2016).

## Supplementary Material

Supplementary Information

## Figures and Tables

**Table 1:** Characteristics of QDs via interparticle spacing and its EL performance.

Surface Ligand	Ligand length (nm)	PL peak (nm)	PL QY (%)	Turn-on voltage @ 1 cd/m^2^ (V_on_)	Maximum luminance (cd/m^2^)	Current efficiency @ Max luminance (cd/A)	External quantum efficiency @ Max luminance (%)
OA-capped QDs	1.7	532	82	2.5	57,000	5.1	1.31
Br-capped QDs	0.1	537	85	3.0	71,000	6.4	1.65
