# Supplementary material for: Efficient exciton generation in atomic passivated CdSe/ZnS quantum dots light-emitting devices
Source: Sci Rep. 2016 Sep 30;6:34659. doi: 10.1038/srep34659 (PMC5043346; doi:10.1038/srep34659)
Supplement: Supplementary Information [file srep34659-s1.doc]

Supplementary Information

Efficient exciton generation in atomic passivated CdSe/ZnS quantum dots light-emitting devices

Byoung-Ho Kang^1,+^, Jae-Sung Lee^2,+^, Sang-Won Lee^2^, Sae-Wan Kim^2^, Jun-Woo Lee^2^, Sai-Anand Gopalan^2^, Ji-Sub Park^2^, Dae-Hyuk Kwon^3^, Jin-Hyuk Bae^2^, Hak-Rin Kim^2^, and Shin-Won Kang^2,*^

*^1^Center for Functional Devices Fusion Platform, Kyungpook National University, Sankyuk-dong, Bukgu, 702-701 Daegu, Republic of Korea*

*^2^School of Electronics Engineering, College of IT Engineering, Kyungpook National University, Sankyuk-dong, Bukgu, 702-701 Daegu, Republic of Korea*

*^3^Department of Electronic Engineering, Kyungil University, Buho-ri, Hayang-eup, 712-701 Gyeongsan-si, Republic of Korea*

^*^Corresponding author. Tel.: 82 53 950 6829, Fax: 82 53 950 7932, E-mail: [swkang@knu.ac.kr](mailto:swkang@knu.ac.kr)

^+^These authors contributed equally to this work.

**1. Component analysis of surface modulated CdSe/ZnS QDs.**

In order to verify the coordination of the Br^-^ anions with the CdSe/ZnS QD surfaces, we analyzed their energy dispersive spectroscopy (EDS) spectra. The atomic percentage of the Br^-^ anion is calculated to be approximately 13%, as shown in Fig. S1. This result confirm that the Br^-^ anion was effectively attached to the QDs surface via electrostatic interaction.

**
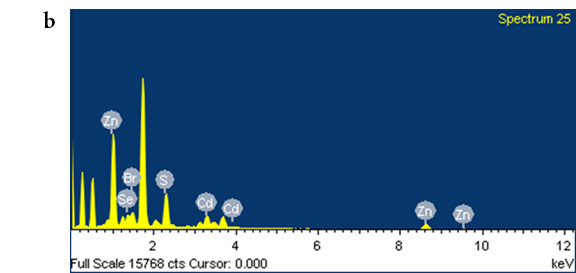
**

**Figure S1. Component analysis of CTAB treated CdSe/ZnS QDs films.** (**a**) FT-IR spectra of OA-capped QDs and Br-capped QDs. (**b**) EDS result for Br-capped QDs.

**2. Surface morphology of CdSe/ZnS films after CTAB treatment.**

**
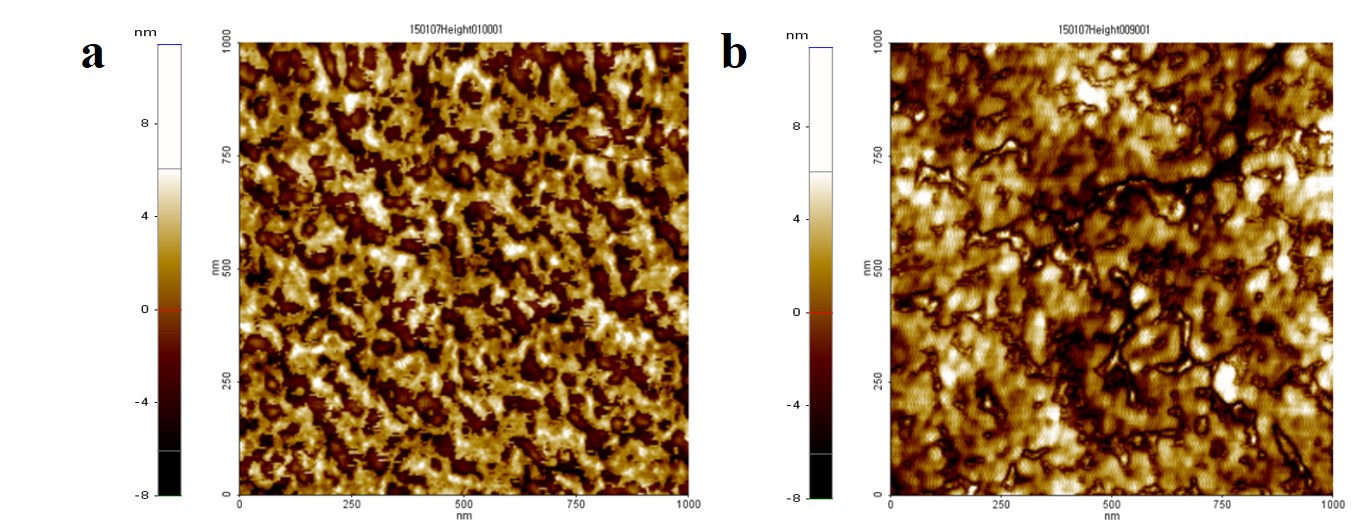
**

**Figure S2.** **Surface morphology results of the EML.** (**a**) OA-capped QDs film. (**b**) Br-capped QDs film.

An atomic force microscope (AFM) was used to measure the surface roughness and determine the film packing density of the EML. The OA-capped QD film has an average and maximum roughnesses of 3.1 nm and 11.9 nm, as shown in Fig. S2a. However, the Br-capped QD film has average and maximum roughnesses of 2.4 nm and 9.8 nm, lower than those of the OA-capped QD layer, as shown in Fig. S2b. These results show that the Br-capped QDs exhibit a more morphologically homogeneous state than the OA-capped QDs because of reduced interparticle spacing.

**3. Photoluminescence quantum yield (PL QY) of CdSe/ZnS QDs after CTAB treatment.**

The relative photoluminescence quantum yield (PL QY) of the QDs was measured by comparing their PL intensities with those of a primary standard dye solution (Rhodamine 6G) at the same optical density (0.05), at the 450 nm excitation wavelength^1^. The Br-capped QDs exhibit a higher PL QY than the OA-capped QDs, as shown in Fig. S3. These results show that Br^-^ anion treatment results in a more efficient thin film emissive layer than is achieved with OA-capped QDs.

**Figure S3. The PL QY of CdSe/ZnS QDs after CTAB treatment.**

**4. CTAB treatment of TOP-capped CdSe/ZnS QDs films.**

In order to investigate the effect of surface modulation with specific organic chains, we tested TOP-capped QDs and also used CTAB as a solid-state film. In this case, the luminance and efficiency of the QLEDs were decreased due to low nucleophilic substitution reactivity. In the reaction between CTAB and TOP, the ammonium cation of CTAB and phosphate from TOP are likely to lead to an unstable ammonium phosphate coupling. As a result, the initial current efficiency is generally high due to charge accumulation in the low current density region. However, the unstable ammonium phosphate causes auger-mediated decay in the device.


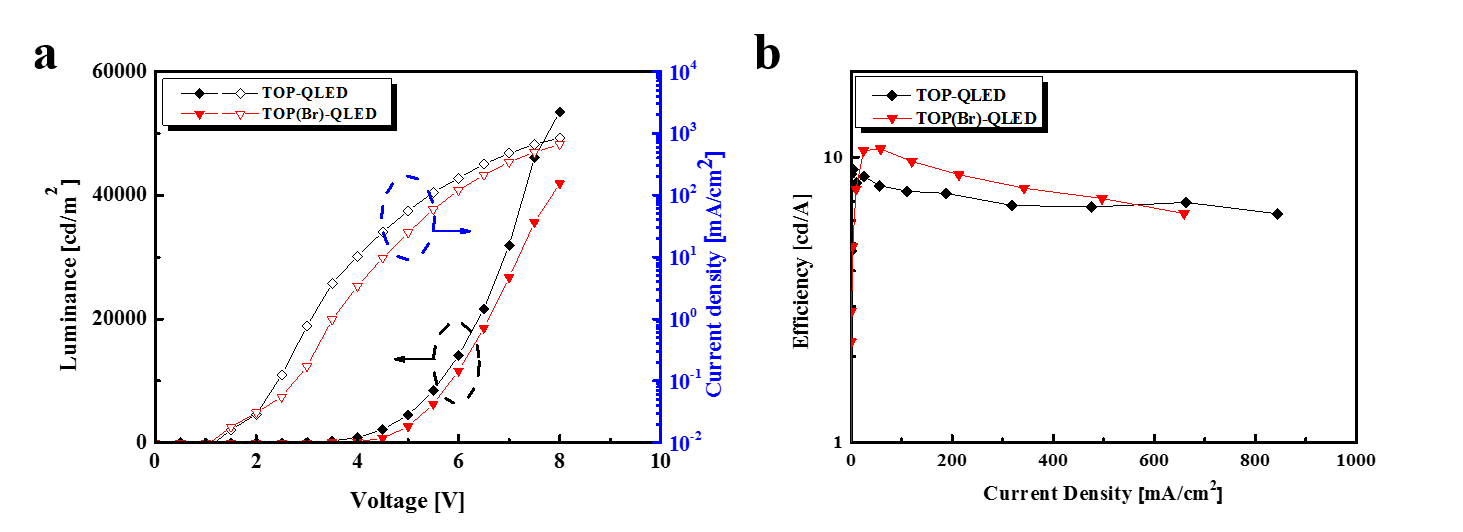


**Figure S4.** **Performance evaluation results for the TOP-capped QLEDs.** (**a**) voltage-luminance and voltage-current density. (**b**) current density-current efficiency.

**5. Synthesis of Gradient green CdSe/ZnS Quantum Dots.**

Green CdSe/ZnS QDs with chemical-composition gradients were prepared according to a method reported in the literature^2^. In a typical synthesis, 0.1 mmol of cadmium oxide (CdO, 99.99%, Sigma-Aldrich), 4 mmol of zinc acetate (Zn(Ac)_2_, 99.99%, Sigma-Aldrich)and 5 ml of oleic acid (OA, 90%, Sigma-Aldrich) were placed in a 50 ml flask and heated to 150 °C in flowing high-purity argon (Ar) for 30 min. Then, 15 ml of 1-octadecene (1-ODE, 90%, Sigma-Aldrich) was added to the 3-neck flask and the temperature was increased to 300 °C. A stock solution containing 0.2 mmol of selenium (Se, 99.99%, Sigma-Aldrich) and 3.5 mmol of sulfur (S,99.98%, Sigma-Aldrich) in 2 ml of trioctylphosphine (TOP, 90%, Sigma-Aldrich) was quickly injected into the 3-neck flask. The reaction temperature was maintained for 10 min and then the flask was cooled to room temperature. Then, the synthesized QDs were purified by adding solution of toluene and ethanol. The mixture was centrifuged at 3,000 rpm for 10 min to separate the QDs via precipitation. The supernatant liquid phase was decanted to remove excess reagent. Subsequently, the QDs were re-dispersed in non-polar toluene solution (10 mg/ml). To determine the characteristics of the CdSe/ZnS QDs, we measured their UV-Vis spectra, PL spectra and collected transmittance electron microscopy (TEM) images as shown in Fig. S5.

**
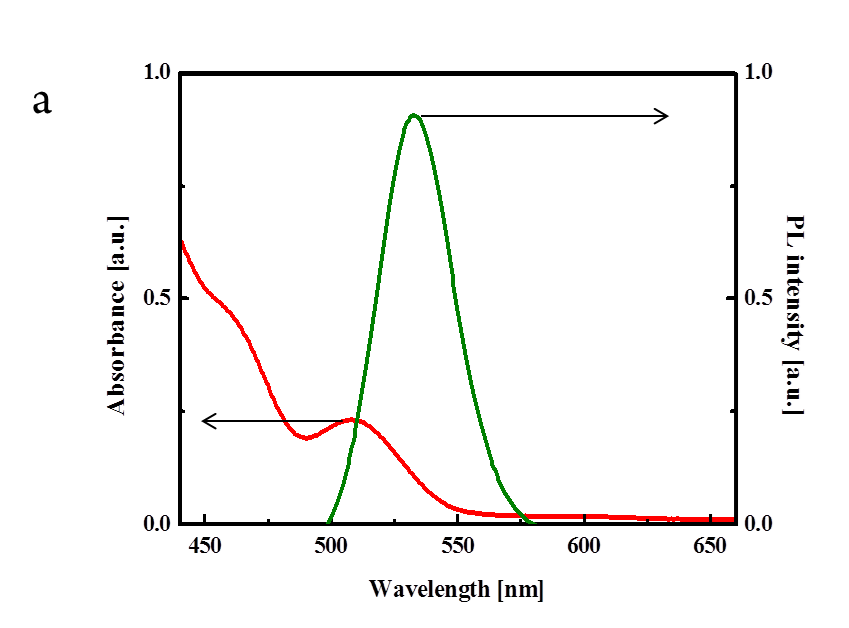
**
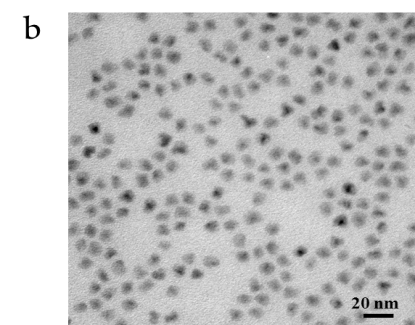


**Figure S5. The characteristics of the synthesized green QDs.** (**a**) UV-Vis absorption and PL spectra. (**b**) TEM image.

**6. Synthesis of ZnO nanoparticles.**

A variant of the sol-gel method was used to synthesize ZnO nanoparticles (NPs) in an alcohol solution^3-6^. The solutions were prepared using 2.46 g of zinc acetate dehydrate (Zn(Ac)_2_·2H_2_O, 99%, AR grade, Sigma-Aldrich) and 0.96 g of potassium hydroxide (KOH, Duksan Pharmaceutical Co. Ltd.) dispersed in 110 ml and 50 ml of methanol, respectively. All chemicals were used as received without further purification. The Zn(Ac)_2_·2H_2_O solution was placed in a 200 ml flask and heated to 60°C and the KOH solution was added dropwise (1ml/sec). The mixture was stirred at 60 °C for 60 min and allowed to cool. To obtain uniform ZnO NPs, we performed the necessary aging process by adding 2-propanol and hexane overnight^7^. The ZnO NPs were then precipitated via centrifuging at 3,000 rpm and re-dispersed in ethanol (30 mg/ml). To determine the ZnO NPs’ characteristics, we measured their UV-Vis spectra and TEM images as shown in Fig. S6.


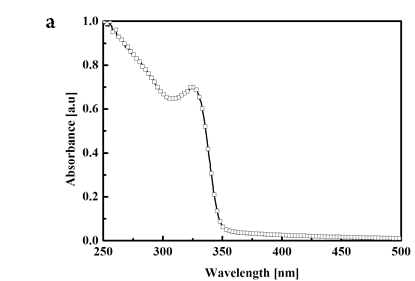

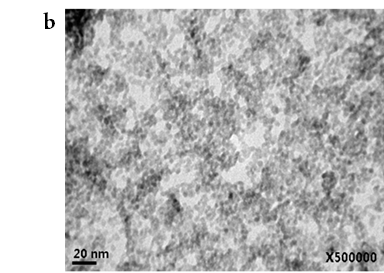


**Figure S6. The characteristics of the synthesized ZnO NPs.** (**a**) absorption spectra. (**b**) TEM image.

**References.**

1. Lee, K.-H. *et al*. Over 40 cd/A Efficient Green Quantum Dot Electroluminescent Device Comprising Uniquely Large-Sized Quantum Dots. *ACS Nano.* **8**, 4893–4901 (2014).

2. Bae, W. K. *et al.* Highly efficient green-light-emitting diodes based on CdSe@ZnS quantum dots with a chemical-composition gradient. *Adv. Mater.* **21**, 1690-1694 (2009).

3. Meulenkamp, E. A. Synthesis and growth of ZnO nanoparticles. *J. Phys. Chem. B.* **102**, 5566-5572 (1998).

4. Asok, A., Mayuri, N., Gandhi, N. & Kulkarni, A. R. Enhanced visible photoluminescence in ZnO quantum dots by promotion of oxygen vacancy formation. *Nanoscale*. **4**, 4943-4946 (2012).

5. Pacholski, C., Kornowski, A. & Weller, H. Self-assembly of ZnO: from nanodots to nanorods. *Angew. Chem., Int. Ed.* **41**, 1188-1191 (2002).

6. Kwak, J. *et al.* Bright and efficient full-color colloidal quantum dot light-emitting diodes using an inverted device structure. *Nano. Lett.* **12**, 2362-2366 (2012).

7. Sun, D. et al. Purification and stabilization of colloidal ZnO nanoparticles in methanol. *J. Sol-Gel Sci. Technol.* **43**, 237-243 (2007).
